# Direct Oxidation of *Hibiscus cannabinus* Stalks to Vanillin Using CeO_2_ Nanostructure Catalysts

**DOI:** 10.3390/molecules28134963

**Published:** 2023-06-24

**Authors:** Anita Ramli, Nur Akila Syakida Idayu Khairul Anuar, Nur Aielia Amira Bakhtiar, Normawati Mohamad Yunus, Alina Rahayu Mohamed

**Affiliations:** 1 HICoE Centre for Biofuel and Biochemical Research, Institute of Self-Sustainable Building, Universiti Teknologi PETRONAS, Seri Iskandar 32610, Perak, Malaysia; nur_18003521@utp.edu.my (N.A.S.I.K.A.); nur_21001096@utp.edu.my (N.A.A.B.); 2 Fundamental and Applied Sciences Department, Universiti Teknologi PETRONAS, Seri Iskandar 32610, Perak, Malaysia; normaw@utp.edu.my; 3 Centre of Research in Ionic Liquids (CORIL), Institute of Contaminant Management for Oil and Gas, Department of Fundamental and Applied Sciences, Universiti Teknologi PETRONAS, Seri Iskandar 32610, Perak, Malaysia; 4 Faculty of Chemical Engineering & Technology, UniMAP, Complex of Academics Jejawi 3, Jejawi, Arau 02600, Perlis, Malaysia; alina@unimap.edu.my

**Keywords:** direct oxidation, vanillin, microwave irradiation, CeO_2_ nanostructured catalyst

## Abstract

Biomass lignin can be used to produce vanillin through an oxidation process. Although its purity is high, the processing time and separation efficiency are not ideal. This research aims to produce vanillin directly from Kenaf stalks without separating the lignin first from the lignocellulosic biomass. This method is greener because it does not require the separation of cellulose and hemicellulose from the biomass, thus minimizing the use of acid and alkaline solutions and saving time. A high oxygen storage capacity and release capacity of ceria as an oxidation catalyst contribute to the reversable redox properties between Ce^4+^ and Ce^3+^ in ceria lattice. Cerium oxide nanostructures were synthesized using a hydrothermal method treated under alkaline NaOH, followed by drying at 120 °C for 16 h and calcining at different temperatures between 400 and 600 °C for the direct oxidation of Kenaf stalks to vanillin under microwave irradiation. The catalysts were characterized for their physicochemical properties using XRD, N_2_ adsorption–desorption isotherms and TEM. All synthesized CeO_2_ nanostructures showed the presence of diffraction peaks assigned to the presence of cubic fluorite. The N_2_ adsorption–desorption isotherms showed that all catalysts possess a Type IV isotherm, indicating a mesoporous structure. The TEM image shows the uniform shape of the CeO_2_ nanostructures, while HRTEM images show that the CeO_2_ nanostructures are single-crystalline in nature. All catalysts were tested for the direct oxidation of Kenaf stalks using H_2_O_2_ as the oxidizing agent in temperatures ranging from 160 to 180 °C for 10–30 min with 0.1–0.3 g catalyst loading under 100–500 W of microwave irradiation. The CeO_2_-Nps-400 catalyst produced the highest vanillin yields of 3.84% and 4.32% for the direct oxidation of Kenaf stalks and extraction of lignin from Kenaf stalks, respectively. Compared to our earlier study, the highest vanillin yields of 2.90% and 3.70% for direct biomass and extracted lignin were achieved using a Ce/MgO catalyst.

## 1. Introduction

Grand View Research, Inc recently reported that by 2025, the global vanillin market size is expected to reach USD 724.5 million [1]. According to Martău et al. [2], the rising consumption of food and beverage products has been a significant driver of vanillin mar-ket growth globally. Vanillin (4-hydroxy-3-methoxybenzaldehyde), which was first isolated from vanilla in 1816, is the major flavor constituent of vanilla [3]. Currently, cured vanilla pods are the main source of natural vanillin. Because of its sweet smell, vanilla has been used primarily as a flavoring in confectionery, food products, beverages, ice cream and perfume [4]. Aside from its appealing aroma, vanillin and vanilla extracts have also been reported to possess various health benefits, such as antioxidant, anti-mutagenic and hypolipidemic activities, and have considerable potential as food preservatives and anticarcinogens [5].

Vanillin consists of an aromatic compound and two reactive functions that can be modified chemically because the aldehyde and phenol are more reactive than the methoxy group [6]. Thus, vanillin can be considered a valuable bifunctional compound for preparing various products using raw resources such as lignin, clove, turmeric, rice bran and corn. Synthetic vanillin obtained from guaiacol is highly regio-selective towards the para position, which avoids side products, simplifying its separation, which is a significant advantage [5]. However, the guaiacol route depends on petroleum-derived compounds. The demand for chemicals derived from renewable resources such as biomass lignin has increased remarkably due to the depletion of fossil fuels and serious concerns about climate change [1].

Lignin is a promising resource for producing a variety of aromatic chemicals and materials because of its unique structure as a complex phenolic polymer with randomly crosslinked C9 units [7]. Sangnak et al. [8] reported the depolymerization of lignin using a batch setup reactor, achieving a 5.91% vanillin yield at 80 °C for 1.5 h under 1.5 g of CuO/Al_2_O_3_ catalyst and a lignin concentration of 0.25 g/L in an alkaline medium of NaOH. The maximum obtained vanillin of 105 mg/g (12.5 wt%) with 85% selectivity was achieved using 20 wt% commercial alkaline lignin catalyzed by 5wt% [BMIM][FeCl_4_] ionic liquid in water medium, and the oxidation reaction was carried out at room temperature for a reaction duration of up to 4 h [9]. On the other hand, Zhou et al. [10] reported the liquid-phase catalytic aerobic oxidation of lignin-based vanillyl alcohol executed in a 50 mL batch autoclave at 180 °C for 2 h in the presence of a 1.0 MPa O_2_ supply in ethyl acetate solvent with the addition of 0.1 g of LaFeO_3_ perovskite catalyst. The catalytic performance yielded 33% vanillin with a 100% conversion rate of vanillyl alcohol. 

The effective extraction or separation of lignin with high purity and a less condensed structure from lignocellulose is crucial for lignin valorization [11]. The extraction of lignin from lignocellulosic biomass requires the use of concentrated mineral acids, which also result in the degradation and depolymerization of the lignin. On the other hand, the efficiency of alkaline hydrolysis is very low. Furthermore, both acid and alkaline hydrolysis require the use of concentrated acid/alkaline and are difficult processes [12]. Thus, Qu et al. [13] reported the direct production of vanillin from wood particles using copper oxide with peroxide as the oxidizing agent under microwave irradiation. They reported vanillin and vanillic acid yields of 8.5% at 200 °C for 10 min. Our initial work on employing Ce and Zr catalysts supported by MgO to directly oxidize Kenaf stalks to produce vanillin indicates that Kenaf stalks using a Ce/MgO catalyst and microwaves give a 2.90 yield of vanillin, while bare MgO and Zr/MgO do not give any vanillin product. Since ceria shows noteworthy performance, this research aims to produce vanillin directly from Kenaf stalks without separating the lignin first from the lignocellulosic biomass, using different nanostructures of ceria to investigate its catalytic performance.

CeO_2_ is a well-known rare earth material with highly desirable chemical and physical properties. In addition, ceria’s exceptional reversible redox properties between Ce^4+^ and Ce^3+^ contribute to its high oxygen storage capacity (OSC) and release capacity [14]. The different shapes of CeO_2_ produce noteworthy specific crystal facets that display different reactivities in various catalytic processes. So far, no research has been reported on the use of CeO_2_ in the oxidation of lignocellulosic biomass to vanillin. *Hibiscus cannabinus*, known as Kenaf, is an annual fiber plant and one of the hard-wood species belonging to the Malvaceae Hibiscus family native to South Asia. Kenaf has high potential as a source of lignin to produce natural vanillin due to its high syringyl to guaiacyl ratio in the aromatic composition of lignin. Furthermore, this plant is also easy to handle, exhibits excellent adaptability compared to other fiber crops and yields three to five times as much fiber as southern pine [1]. Thus, in this project, CeO_2_ was synthesized in three different nanostructures (nanorod, nanocube and nanoparticle) and calcined at different temperatures to produce various catalysts for the direct oxidation of Kenaf stalks to vanillin in reactions with different parameters.

## 2. Results and Discussion

### 2.1. Characterization of Catalyst

**Figure 1:**
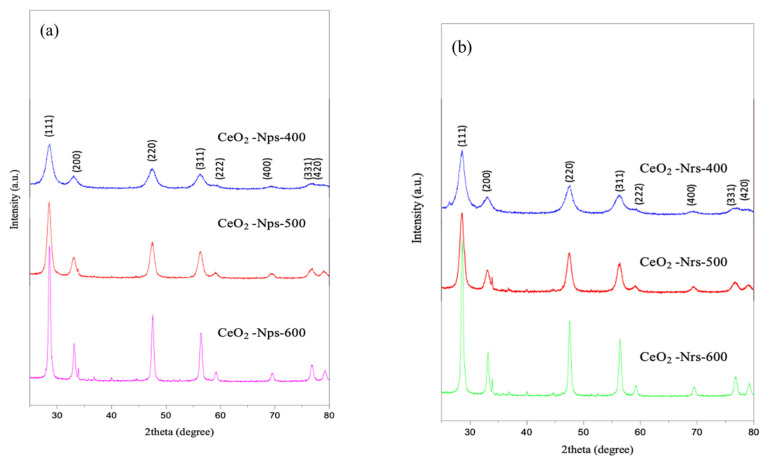

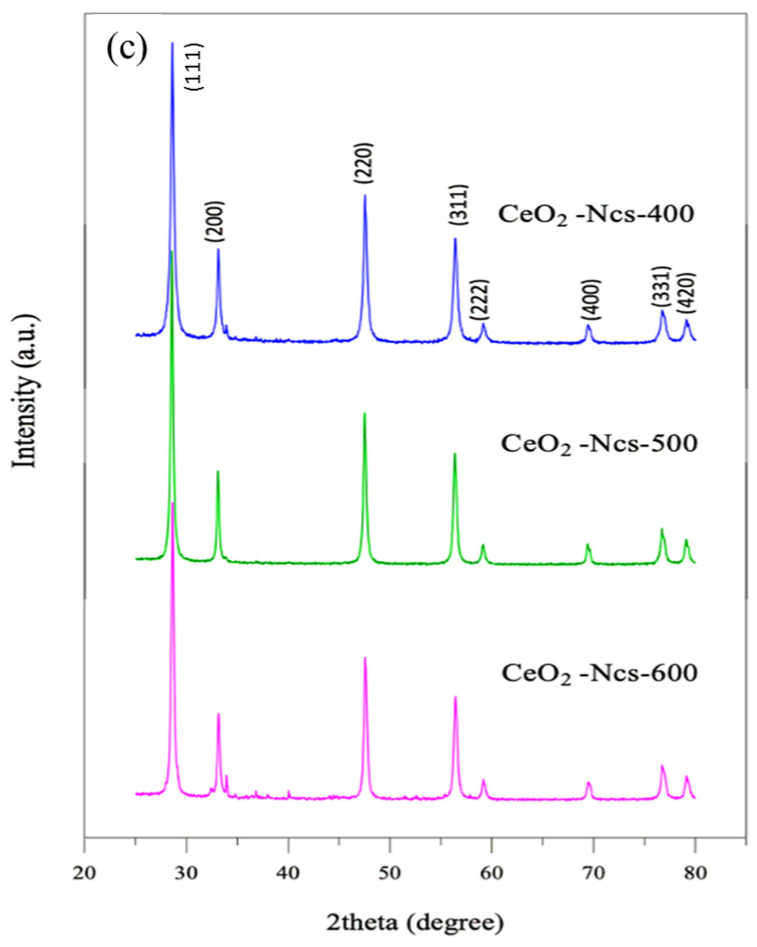
XRD patterns for (**a**) CeO_2_ nanoparticles, (**b**) CeO_2_ nanorods and (**c**) CeO_2_ nanocubes calcined at 400 °C, 500 °C and 600 °C for 2 h.

Figure 1 shows the X-ray diffractograms of synthesized CeO_2_ nanoparticles, nanorods and nanocubes calcined at 400, 500 and 600 °C for 2 h. All peaks in the XRD patterns can be perfectly indexed to ICSD: 81-0792, and based on the full width at the half-maximum (FWHM) of the diffraction peak (111), the crystallite sizes of CeO_2_ nanostructures calcined at different temperatures were estimated via the Scherrer equation and are summarized in Table 1. The result is in agreement with Meng et al. [15], who reported that the presence of diffraction peaks at 2θ = 28.53, 33.07, 47.46, 56.31, 59.06, 69.38, 76.66, and 106 79.03° can be assigned to the (111), (200), (220), (311), (222), (400), (331) and (420) planes. Furthermore, all Bragg reflections for all the CeO_2_ nanostructures agree well with those of standard CeO_2_, which indicates the formation of pure-phase CeO_2_ in a cubic fluorite structure [16]. 

According to the XRD pattern for all catalysts, the diffraction peaks of CeO_2_ nanostructures calcined at 400 °C are quite broad, indicating that the catalyst’s crystallite sizes are petite [17]. On the other hand, the diffraction peaks become sharper and the line broadening decreases as the calcination temperature increases to 500°C, which is attributed to the increased product size and demonstration of good crystallinity. These observations can be related to Table 1, which shows the crystallite size of all the CeO_2_ nanostructures corresponding to the (111) plane with different calcination temperatures at 400, 500 and 600 °C for 2 h. As calcination temperature increased from 400 to 600 °C, crystallite size gradually increased for all CeO_2_ nanostructure samples. This result can be confirmed by a study by Gharagozlou [18], where they suggested that during the calcination process, two or more particles tend to fuse together by melting through their surfaces. As a result, the substantial increase in crystallite size as calcination temperature rises indicates the formation of larger particles. It can also be observed that CeO_2_-Nps-400 has the smallest crystallite size, followed by CeO_2_-Nrs-400 and CeO_2_-Ncs-400.

**Figure 2:**
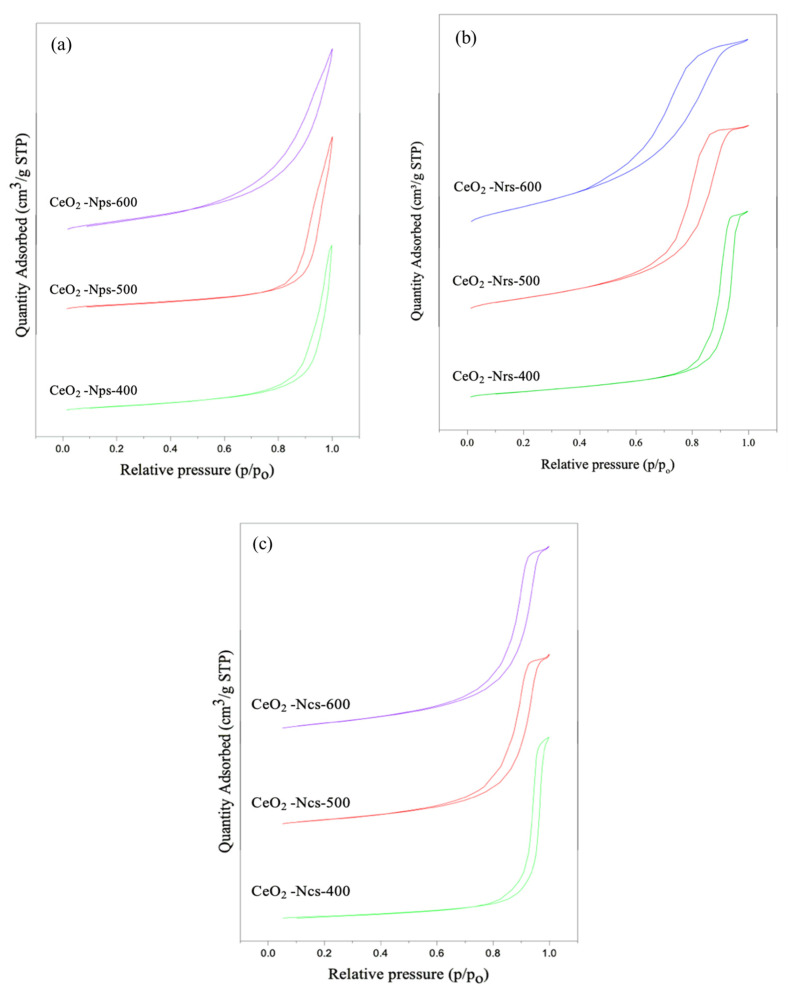
N_2_ adsorption–desorption of for (**a**) CeO_2_ nanoparticles, (**b**) CeO_2_ nanorods and (**c**) CeO_2_ nanocubes.

Figure 2 shows the isotherm plot for the CeO_2_ nanostructures with different calcination temperatures of 400, 500 and 600 °C. According to the classification of N_2_ adsorption–desorption made by the International Union of Pure and Applied Chemistry (IUPAC), the isotherm plots for all catalysts exhibit Type IV isotherms with significant H1 hysteresis loops [19]. Therefore, all of the CeO_2_ nanoparticles were classified as mesoporous materials. Table 2 shows the textural properties of the CeO_2_ nanostructures where the surface area, pore volume and average pore size of the synthesized CeO_2_ nanoparticles increase with increasing the calcination temperature from 400 to 600 °C. The CeO_2_ nanoparticles calcined at 400 °C possessed the largest BET surface area of 66.1 m^2^/g, three times greater than that calcined at 600 °C. The CeO_2_ nanorods calcined at 400 °C possessed the largest BET surface area of 57.8 m^2^/g, which was two times greater than that of CeO_2_ nanorods calcined at 600 °C. The CeO_2_ nanocubes calcined at 400 °C possessed the largest BET surface area of 22.5 m^2^/g, which was two times greater than that of CeO_2_ nanocubes calcined at 600 °C.

According to Chowdhury et al. [20], the BET surface areas of samples decreased as the calcination temperature increased, which may have been caused by the development of crystalline CeO_2_ that blocked the pores. Furthermore, the greater the calcination temperature, the wider the pore diameter because the residual moisture in the pores evaporates, which could be due to the formation of crystalline CeO_2_ blocking the pores. Moreover, the higher calcination temperature results in a larger pore diameter because the remaining moisture in the pores evaporates [19]. Due to significant agglomeration, the pore volume of the catalyst decreased as the calcination temperature increased [18,21]. The surface area and crystallite size of all catalysts showed a clear contradictory trend with each other, which agrees with the findings stated by Chowdhury et al. [20]. Their study reported that a bigger total surface area of the materials would result in larger interparticle porosity or voids between particles, thus reducing the crystallite size of the material.

**Figure 3:**
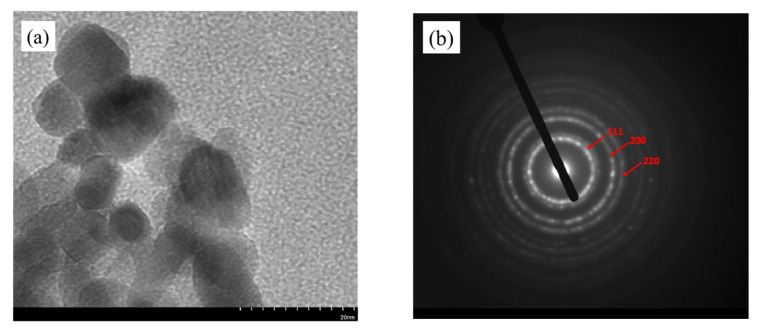

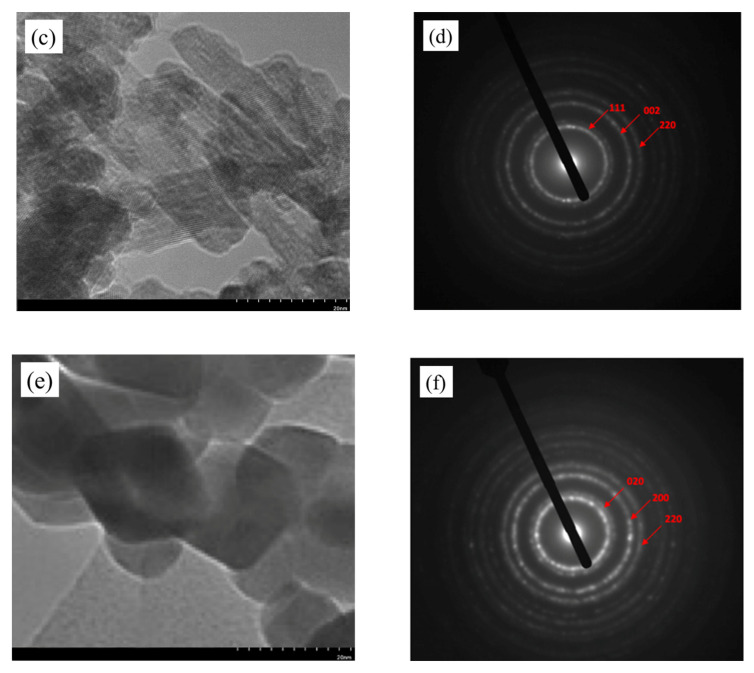
TEM (**a**) and HRTEM (**b**) images of CeO_2_-Nps-400. TEM (**c**) and HRTEM (**d**) images of CeO_2_-Nrs-400. TEM (**e**) and HRTEM (**f**) images of CeO_2_-Ncs-400.

Only CeO_2_ nanostructures calcined at 400 °C were further characterized using TEM to study their morphology and structure in catalyst analysis since they produced the highest amounts of vanillin compared to other catalysts calcined at 500 and 600 °C, with vanillin yields of 0.45, 0.39 and 0.36% for CeO_2_-Nps-400, CeO_2_-Nrs-400 and CeO_2_-Ncs-400, respectively. Figure 3a shows the TEM image of the as-obtained uniform CeO_2_ nanoparticles. The TEM images show loose agglomeration of CeO_2_ nanoparticles, implying that particles could be separated from one another [22] and suggesting that the size of CeO_2_ particles is dispersed in a very narrow range that consists of particle-like networks, with the largest particle sizes being ~15–20 nm. The HRTEM image in Figure 3b shows clear (111), (200), and (220) lattice fringes, revealing that the CeO_2_ nanoparticles are dominated by a truncated octahedral shape enclosed by the [111] and [100] facets [23]. The TEM image reveals ceria in the rod-like morphology as depicted in Figure 3c for CeO_2_ nanorods. Figure 3d depicts the HRTEM image of a CeO_2_ nanorod that shows three kinds of lattice fringe directions attributed to (111), (002), and (220), as observed for the nanorods. The nanorods show a 1D growth structure with a preferred growth direction along [110] and are enclosed by the (220) and (200) planes, which is similar to the case of CeO_2_ nanorods prepared under similar hydrothermal conditions by Lin et al. [24]. The CeO_2_ nanorods are reported to promote the exposure of [110]. Figure 3e reveals the TEM image of the CeO_2_ nanocubes. The HRTEM image in Figure 3f displays the clear (200) and (220) lattice fringes, implying that the CeO_2_ nanocubes are only enclosed by the (200) planes and that ceria nanocubes promote the exposure of [100] facets. Such a cube shape was rarely observed for CeO_2_ in previous studies [25]. From the HRTEM images shown in Figure 3, we can also see that the as-obtained CeO_2_ nanoparticles, nanorods and nanocubes are of single-crystalline nature. 

### 2.2. Extraction of Lignin

**Figure 4:**
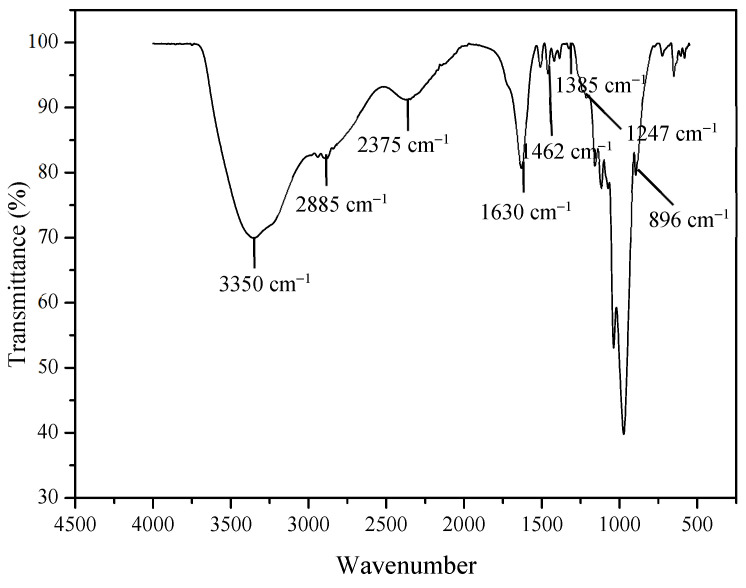
FTIR spectra of dried Kenaf stalk lignin.

The lignin yield obtained from Kenaf after alkaline hydrolysis was 22.5%. The amount of lignin content from Kenaf ranged from 19.2% to 21.2%. Softwoods and hardwoods contribute 25–35% and 18–25% of lignin, respectively, to the dry weight of wood. The lignin content was isolated using kraft lignin of Kenaf, which gives a yield of 20.8% as reported by Mohamad Aini et al. [26]. On the other hand, research performed by Song et al. [27] on the extraction of lignin from Kenaf resulted in 16.76% lignin from raw Kenaf using an acid hydrolysis method. The infrared spectrum of the extracted lignin is depicted in Figure 4. The broad peak at 3350 cm^−1^ corresponds to the stretching vibration of O–H bonds [28] and the band at 2886 cm^−1^ is attributable to the C–H stretching vibration in the methyl and methylene groups [29]. The C–H bending vibration in the methyl groups can be assigned to the band at 1462 cm^−1^ [30]. The band of 1650–1590 was due to C=C aromatic vibrations allocated to the presence of lignin. The bands for C=C stretching vibration in the syringyl propane unit (S) and the guaiacyl propane unit (G) were centered at 1385 cm^−1^ and 1247 cm^−1^, respectively [31]. The band at 1037 cm^−1^ can be assigned to the C–O deformation in secondary and primary alcohol or aliphatic ethers [32,33]. An absorption band appeared at 897 cm^−1^ attributable to C–O–C stretching at the β– (1, 4)–glycosidic linkage in cellulose [34].

### 2.3. Catalyst Screening

**Figure 5:**
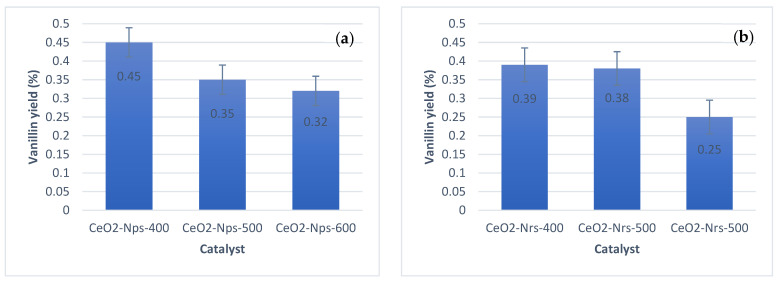

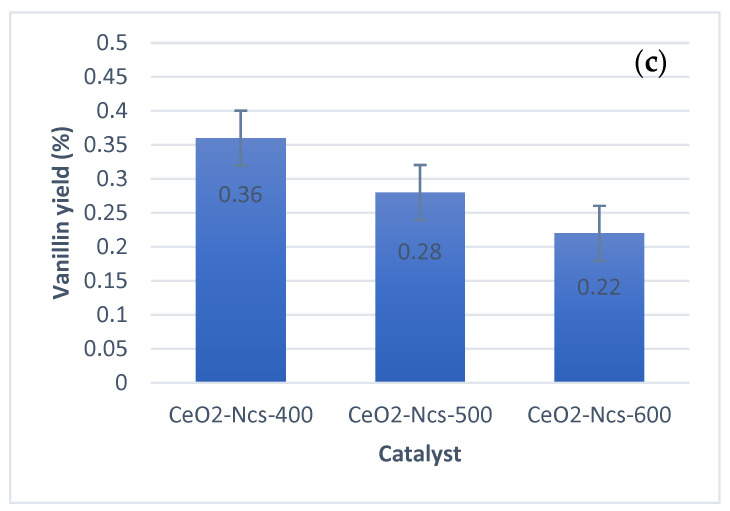
Screening of CeO_2_ (**a**) nanoparticles, (**b**) nanorods and (**c**) nanocubes as catalyst for production of vanillin.

The figure below shows the catalyst screening performance using CeO_2_ nanostructures and catalytic performance of the catalysts at fixed reaction conditions, i.e., a catalyst loading of 0.2 g at 180 °C for 20 min under microwave irradiation. Using high-performance liquid chromatography (HPLC), the vanillin yield was determined and evaluated by comparing the derived and standard vanillin retention times. The linear equation derived from the calibration curve determines the quantity of vanillin produced by directly oxidizing Kenaf stalks. Detailed information on HPLC interpretation results will be discussed in Section 2.4. The result illustrated in Figure 5 shows that the vanillin conversion product has the same variation tendency at a calcination temperature of 400 °C, where the highest vanillin yield was recorded, and subsequently decreased with increasing calcination temperature of the catalyst at 500 °C and 600 °C. 

As calcination temperature increased, the catalysts demonstrated a different degree of influence on the dispersion state, structure of the active compound and pore structure, including their crystallinity properties as portrayed in the XRD and BET results. All ceria nanostructures calcined at 400 °C, especially CeO_2_-Nps-400, show an increasing pattern of vanillin yield, with 0.45, 0.39 and 0.36% for CeO_2_-Nps-400, CeO_2_-Nrs-400 and CeO_2_-Ncs-400, respectively. However, vanillin production shows a slightly decreasing pattern as calcination temperature increases. This suggests that the calcination temperature causes catalyst aggregation, which changes the catalyst’s textural properties [35]. When the calcination temperature increased from 500 to 600 °C, the pores of the catalyst collapsed and were blocked; additionally, the catalyst pores were sintered. All of the mentioned factors can eventually cause losses of catalytic activity and affect the effectiveness of the catalyst surface as reactions occur on the catalyst surface or/and pores [36].

The XRD and BET results also show an increasing pattern in crystallite size as well as a decreasing pattern in surface area for all ceria nanostructures as calcination temperature increased. The decreasing crystallite size of ceria leads to higher surface area, which also leads to the formation of more oxygen vacancies. The bigger particle size would result in lattice expansion in the crystal structure. The expansion of the lattice would decrease its oxygen release and reabsorption capabilities [37], which is also related to the lower dispersion rate. All CeO_2_ nanostructures calcined at 400 °C display a good dispersion state of active components with reaction reactant compared to CeO_2_ nanostructures calcined at 500 and 600 °C. The results obtained demonstrate that the decreasing production of vanillin was due to low degree of catalyst dispersion state and the catalytic activity continued to decrease, as suggested by Chen et al. [38].

This also can relate to the higher surface area of the catalyst. Factors affecting reaction rate also show that high surface area increased the reaction rate compared to the low surface area exhibited by CeO_2_ nanoparticles calcined at 500 and 600 °C. Thus, the catalyst is exposed to many active sites that allow oxidation. As a result, there is a greater chance of particles colliding with active sites, which leads to more successful collisions per second. It is the same case for CeO_2_ nanorods and nanocubes, whereby the catalyst calcined at 400 °C produced more vanillin than the catalysts calcined at 500 and 600 °C. Therefore, CeO_2_-Nps-400, CeO_2_-Nrs-400 and CeO_2_-Ncs-400 were chosen as the best catalysts. The different ceria nanostructures also led to significant changes in catalytic activity, as displayed in the TEM results. Ceria nanoparticles were dominated by the [111] and [100] facets, followed by exposure of [110] for nanorod structure and exposure of [100] facets for nanocubes. Generally, these three low-index facets on CeO_2_ nanostructures follow the reactivity order of [100] ˃ [110] ˃ [111]. This indicates that different facets possess different reactivities in catalytic activity [34].

### 2.4. Catalyst Testing

**Figure 6:**
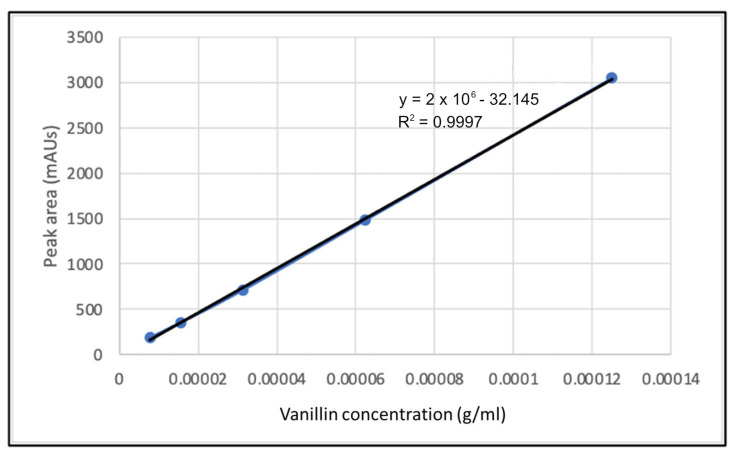
Calibration curve of the vanillin standard at five different concentrations.

Figure 6 shows the calibration curve of the vanillin standard at five different concentrations (1.25 × 10^−4^, 6.25 × 10^−5^, 3.125 × 10^−5^, 1.56 × 10^−5^ and 7.81 × 10^−6^ M) using HPLC. To prepare a calibration curve of vanillin, each working standard solution with different concentrations was injected into the HPLC system. Then, the curve was plotted based on measuring the peak area attained from the injected aliquot of standard vanillin solution with known concentration. This was used to determine the vanillin yield quantitatively. The correlation coefficient, R^2^, of the best-fit line displayed 0.9997, which indicates high accuracy. Therefore, the vanillin yield was determined quantitatively using the calibration curve.

**Figure 7:**
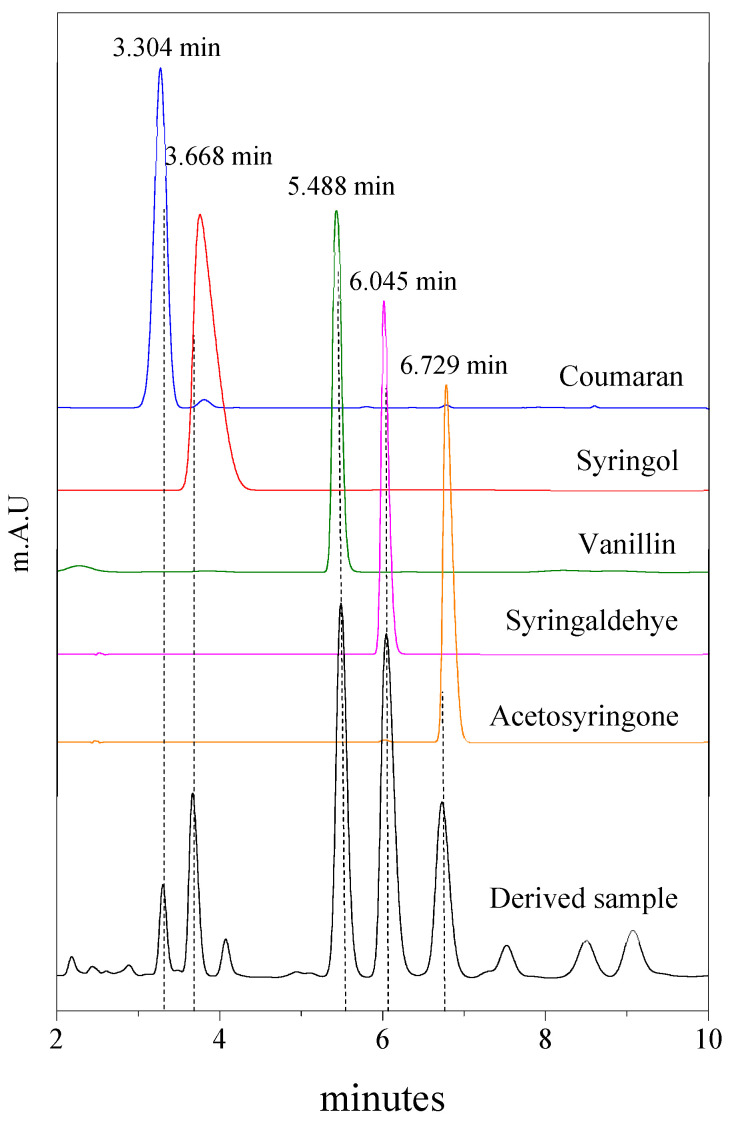
HPLC chromatogram of derived sample and five different standards.

Figure 7 displays the high-performance liquid chromatogram of the derived sample and five different standards (coumaran, syringol, vanillin, syringaldehyde and acetonesyringone) corresponding to the presences of vanillin and other compounds detected in the derived sample with high intensity. When the catalysts were tested in direct oxidation of Kenaf stalks, the liquid produced from oxidation in the presence of CeO_2_ nanostructure catalysts showed the appearance of vanillin peaks observed at the retention of 5.488 min. However, the chromatogram also reveals the presence of four additional compounds with high-intensity peaks that formed at the retention times of 3.304, 3.668, 6.045 and 6.729 min which were attributed to coumaran, syringol, syringaldehyde and acetosyringone, respectively. The vanillin produced from the direct oxidation of Kenaf stalks at 170 °C and ambient pressure could be determined using the linear equation derived from the calibration curve (Figure 6). The vanillin peak shows the most dominant peak detected in the derived sample, followed by syringaldehyde. As reported by other studies, there are many impurities or by-products detected in vanillin produced by the lignin process such as 5-formylvanillin, *para*-coumaric acid, *para*-hydroxybenzaldehyde, syringaldehyde, syringic acid, acetovanillone (4-hydroxy-3-methoxy-acetophenone), vanillic acid and other compounds that were also present in the derived sample, where almost all of the by-product compounds possess aldehyde, alcohol and methoxy groups [6,13,39,40,41,42]. Vanillin derived from lignin still displays large amounts of many different impurities and the purification of vanillin from other phenolic compounds is a challenging task due to the near-identical similarities in chemical structure and properties [43,44].

### 2.5. Optimization of Reaction Conditions for Direct Oxidation of Kenaf Stalks to Vanillin

#### 2.5.1. Effect of Reaction Duration

**Figure 8:**
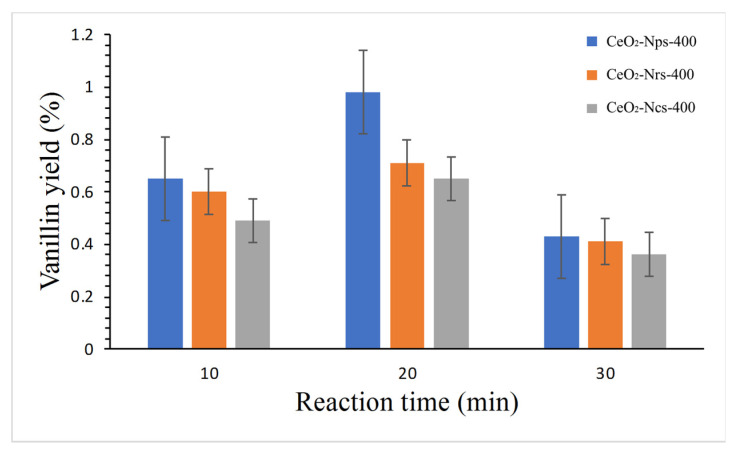
Reaction conditions: 2 g of dried Kenaf stalks, 20 mL of 0.01 N NaOH solution, 0.2 g catalyst, 1 mL of H_2_O_2_ at 180 °C.

The effect of reaction time on vanillin yield is depicted in Figure 8. Generally, under alkaline conditions, vanillin conversion rose with a time increase from 10 to 20 min for all catalysts. Specifically, the CeO_2_-Nps-400 catalyst showed the highest vanillin yield of 1.00% when the reaction was performed for 20 min at 180 °C, while CeO_2_-Ncs-400 and CeO_2_-Nrs-400 gave vanillin yields of 0.65 and 0.72%, respectively. However, when the duration was extended to 30 min, the vanillin yield dropped for all the nanostructures. The above findings suggest that vanillin is particularly unstable under reasonably harsh conditions, where it can be converted to another side product with an extended reaction time. This result agrees with a study by Qu et al. [13] that reported that a 5.1% vanillin yield was obtained with 20 min of reaction duration heated under 180 °C. Japanese cedar wood was directly oxidized with a CuO catalyst combined with peroxide as an oxidizing agent. According to Zhu et al. [28], due to tandem reactions, lengthy reaction durations may increase conversion but decrease selectivity. Khairul et al. [39] reported that a 2.90% vanillin yield was catalyzed by a Ce/MgO catalyst when the reaction was performed for 20 min at 180 °C; however, vanillin yield dropped when the reaction was continued for 30 min. They stated that an extended reaction duration might lead to the over–oxidation of vanillin that formed before the end of the reaction at 30 min, which may also be the optimum reaction conditions for producing other aromatic aldehyde by-products.

#### 2.5.2. Effect of Reaction Temperature

**Figure 9:**
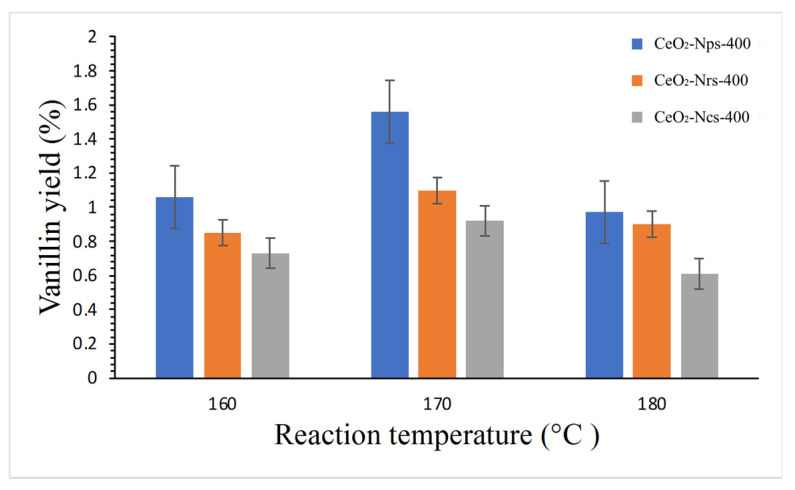
Reaction conditions: 2 g of dried Kenaf stalks, 20 mL of 0.01 N NaOH solution, 0.2 g catalyst 1 mL of H_2_O_2_ under 500 W of microwave heating at 170–180 °C for 20 min.

The impact of reaction temperature on vanillin yield using CeO_2_ nanostructures is shown in Figure 9. Vanillin yield rose when the temperature was raised from 160 °C to 170 °C, but when the temperature was raised to 180 °C, the vanillin yield fell regardless of the catalyst used, which could be attributed to the degradation of vanillin at high temperatures, as reported by Qu et al. [13]. Theoretically, a higher temperature should result in a higher reaction rate, producing more vanillin. However, the faster degradation of vanillin at high operating temperatures contributed to the reduction in vanillin yield, which agrees with the findings of Fengel and Wegener [45]. They claimed that harsh kraft lignin treatment conditions, such as high temperature (180 °C), could result in deeper lignin condensation, resulting in a lower vanillin yield. As we can see, the CeO_2_-Nps-400 showed a high yield of vanillin that gives 1.58%, followed by CeO_2_-Ncs-400 and CeO_2_-Nrs-400, which gave vanillin yields of 1.9 and 1.34%, respectively, under 170 °C reaction temperature. A finding by Ouyang et al. [46] reported that the production of vanillin gives 4.7% from yellow poplar lignin in the presence of Cu^2+^ and Fe^3+^ containing hydroxide as a catalyst in reaction conditions of 170 °C for 20 min under a constant level of O_2_ partial pressure. When the reaction temperature was further increased to 180 °C, they reported that the vanillin yield decreased to 4.3%, which might be due to the degradation of vanillin in high temperatures and under high oxygen pressure.

#### 2.5.3. Effect of Power Output

**Figure 10:**
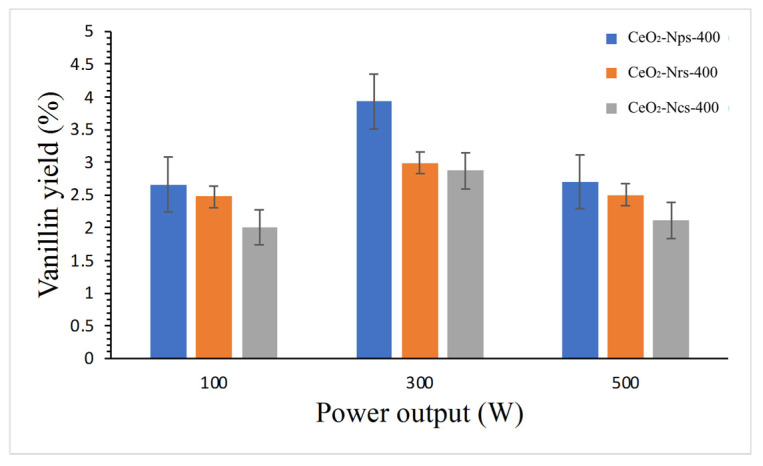
Reaction conditions: 2 g of dried Kenaf stalks, 20 mL of 0.01 N NaOH solution, 0.3 g of catalyst, 1 mL of H_2_O_2_ under 100–300 W of microwave heating at 170 °C for 20 min.

Figure 10 shows the effect of microwave power output on vanillin yield using CeO_2_ nanostructures. An increase in power output from 100 W to 300 W resulted in an increased vanillin yield, which can be related to the more significant amount of converted microwave energy from thermal energy. The vanillin yields obtained under 300 W power output were 4.24%, 3.07% and 2.96% for CeO_2_-Nps-400, CeO_2_-Nrs-400 and CeO_2_-Ncs-400, respectively. According to Zhao et al. [32], there is an increase in vanillin yield when the power irradiation ranges from 50 W to 250 W. This is due to the fact that the intensity of the electromagnetic radiation increases at the same time, resulting in a large amount of heat energy being generated by rapidly alternating electricity compared to conventional heating. A study by Gu et al. [47] proves that the production yield of vanillin improves using the microwave. They reported a 9.94% yield of vanillin was achieved from organosolv lignin in 25 min of reaction time under 200 W of microwave irradiation with the use of mesoporous La/SBA–15 catalyst, which is thirty times greater than under conventional heating. Nevertheless, the vanillin yield fell when the power output was increased to 500 W. This phenomenon might be related to vanillin degradation produced using high power output, which reaches maximum conditions and begins to degrade due to excessive heating from higher power output.

#### 2.5.4. Comparison of Direct Oxidation of Kenaf with Extraction of Lignin

**Figure 11:**
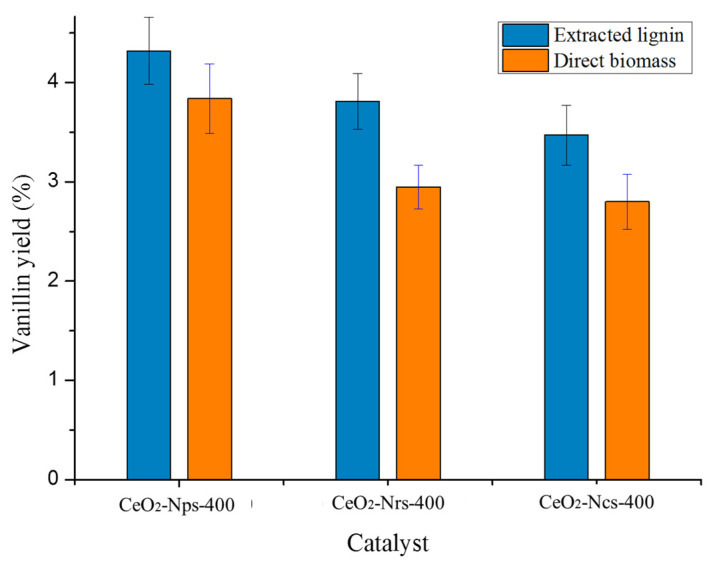
Reaction conditions: 2 g of dried Kenaf stalks and 0.45 g of extracted lignin, 20 mL of 0.01 N NaOH solution, 0.3 g of catalyst, 1 mL of H_2_O_2_, under 300 W of microwave heating at 170 °C for 20 min.

Figure 11 illustrates vanillin production from biomass synthesized with 0.3 g of heterogeneous catalyst treated in a microwave for 20 min at 170 °C under 300 W of microwave power output. The best operating conditions for time, temperature, catalyst loading and microwave power output were selected to produce vanillin directly from biomass and extract lignin from 2 g of Kenaf stalks. According to Figure 11 CeO_2_-Nps-400 was observed producing the highest amount of vanillin for the oxidation of extracted lignin and the direct oxidation of biomass, with vanillin yields of 4.32% and 3.84%, respectively. Due to the chemical structure of cellulose and hemicellulose, we anticipated that only lignin would produce vanillin in the direct oxidation reaction, while cellulose and hemicellulose would produce sugars. Because lignin was isolated as an active ingredient for conversion to vanillin, the extracted lignin demonstrated a higher vanillin yield than direct biomass. Vanillin is produced as a result of the breakdown of aryl ether linkages in β-O-4 bonds and Cα-Cβ cleavage between lignin structures during the oxidation process [48]. Lower vanillin formation resulted from oxidizing biomass without first separating lignin from cellulose and hemicellulose. 

## 3. Materials and Methods

### 3.1. Catalyst Preparation

CeO_2_ nanostructures were synthesized following the method reported by Torrente-Murciano et al. [49]. Typically, to synthesize CeO_2_ nanoparticles, 0.6 g of Ce(NO_3_)_3_·6H_2_O was added to 40 mL of 5 M NaOH solution and stirred for 10 min in a PTFE beaker. Then, the mixture was transferred to a Teflon-lined hydrothermal vessel, sealed and heated to 70 °C for 10 h. At the end of the synthesis duration, the hydrothermal vessel was cooled to room temperature before it was unsealed. The solid was filtered and washed several times with deionized water followed by drying at 120 °C for 16 h and calcination in air at 400 °C, 500 °C and 600 °C for 2 h. The same procedure was repeated to synthesize CeO_2_ nanorods, except the hydrothermal temperature was 100 °C. CeO_2_ nanocubes were synthesized using the same procedure, except the concentration of the NaOH solution used was 15 M and the hydrothermal temperature was 180 °C.

### 3.2. Catalyst Characterization

The crystallinity of the CeO_2_ catalysts was determined using Bruker *X*-ray diffraction (XRD) model X’Pert^3^ Powder and Empyrean (PAN analytical, Billerica, MA, USA). The catalysts were recorded on an *X*-ray Diffractometer system between 2θ values of 20 and 80° with a 227 s/step exposure time and 0.105°/step step size. The surface area and pore size of the catalysts were analyzed using Brunauer–Emmett–Teller (BET) analysis (Micromeritics ASAP 2020, Norcross, GA, USA). The catalysts were degassed at 200 °C for 24 h prior to N_2_ adsorption measurement at −77 °C. The crystallite size of the synthesized CeO_2_ nanoparticles was determined according to Scherrer’s equation: D = kλ/βcosθ. Crystallite size was analyzed using HighScore Plus software (version 3.0), Malvern, UK with an *X*-ray wavelength of CuKα radiation of λ = 1.54 A, where θ is the Bragg angle. β is the full width at half maximum in radians, taken corresponding to the 2θ value at plane (111). The unknown shape factor, k, was assumed to be 0.89, and the reflecting peak at 2θ was chosen for the entire sample. 

### 3.3. Extraction of Lignin and Characterization

Dried Kenaf stalks were broken into 10 cm lengths before being grinded into small particles using a grinder. The extractive compounds such as protein, waxes, resin, fatty acids and lipids were removed from 2 g of dried Kenaf stalks using a mixture of ethanol and water with a (2:1) ratio via Soxhlet extraction for 8 h. The extracted sample was dried in an oven for 24 h at 60 °C. The dried residue was then soaked in an NaOH solution for 5 min with a sample to solvent ratio of (6:100 g/mL). The mixture was stirred and placed into a hydrothermal pressure vessel prior to the cooking process at 121 °C for 2 h to break the bonds between lignin and hemicellulose. The heated mixture was left to cool at room temperature before the separation of solid residue through filtration. The dark brown sample fluid (black liquor) was kept overnight at 4 °C (refrigerator). The black liquor was acidified by H3PO4 (20%) at pH 2. Lignin precipitates into a semi-solid form at around pH 14. The precipitated lignin was then vacuum filtrated using a 0.45 µm pore filter and washed using distilled water and 20% H3PO4. The isolate was dried in an oven at 55 °C for 48 h [39]. The sample was characterized using FTIR (Perkin Elmer Frontier 01, Cheshire, UK) between 500 and 4000 cm^−1^ for 50 scans with a resolution of 4 cm^−1^.

### 3.4. Catalytic Oxidation of Raw Kenaf

Under microwave irradiation, the performance of the synthesized CeO_2_ nanostructures was investigated in the direct oxidation of Kenaf stalks to vanillin. We soaked 2 g of dried Kenaf stalks and 0.45 g of extracted lignin for 24 h in a screw-cap test tube with 20 mL of 0.01 N NaOH solution before transferring to a Teflon tube. After that, 0.2 g of catalyst and 1 mL of H_2_O_2_ were added to the mixture, and the pH was adjusted to pH 11.5 by adding 1 mL of H_2_O_2_. The mixture was heated in a microwave (Milestone, MicroSYNTH MA143) at 160–180 °C for 20 min after being swirled at 600 rpm for 10 s at room temperature using a magnetic stirrer. The liquid was then cooled to room temperature and filtered using filter paper to eliminate any insoluble materials. The residue was washed twice with a 20 mL solution of 0.01 N NaOH. The filtrate was poured into a glass test tube and sealed. Concentrated hydrochloric acid (37%) was added to the filtrate in a 1:2 ratio, agitated for 15 s with a vortex agitator at 5000 rpm, then centrifuged for 15 min at 1000 rpm. The supernatant was then treated with 1:1 ethyl acetate. Vanillin and other low-molecular-weight molecules were extracted into the organic phase. The mixture was agitated for 60 s at 5000 rpm, then centrifuged for 5 min at 1000 rpm, separating the mixture into two phases. The upper phase was placed in a vial. At 50 °C and 400 mbar, excess solvent was evaporated using a rotary evaporator. High-Performance Liquid Chromatography (HPLC, Agilent 1200 series HPLC system) with a UV-VIS detector at a wavelength of 280 nm was used to evaluate the brownish solution containing vanillin. A Hypersil C18 column (particle size, 5 m, 150 4.6 mm inner diameter) was used in the HPLC. The column was heated to 35 °C, and a 20 μL injection volume of acetonitrile: water (1:8 *v*/*v*) containing 1% acetic acid was used as the mobile phase at a flow rate of 2 mL/min. Vanillin (Sigma-Aldrich, St. Louis, MO, USA, purity 99.7%), syringaldehyde (Sigma-Aldrich, purity 98%), acetosyringone (Sigma-Aldrich, purity 97%), coumaran (Sigma-Aldrich, purity 99%) and syringone (Sigma-Aldrich, purity 99%) were used as standards. A calibration curve for vanillin was constructed so that the amount of vanillin produced could be determined quantitatively [13,39].

## 4. Conclusions

CeO_2_ nanostructures were successfully prepared using the hydrothermal method. Their catalytic activity was evaluated in the direct oxidation of Kenaf stalks and lignin extracted from Kenaf stalks under microwave heating using H_2_O_2_ as an oxidizing agent. The CeO_2_ nanostructures calcined at 400 °C were ideal catalysts and hence demonstrated better Kenaf oxidation performance than the CeO_2_ nanostructures calcined at 500 °C and 600 °C. Vanillin yields of 3.84% and 4.32% were achieved from direct oxidation from biomass and extracted lignin from Kenaf stalks, respectively, using the CeO_2_-Nps-400 catalyst at 170 °C for 20 min with catalyst loading of 0.3 g under 300 W of microwave power output.

## Figures and Tables

**Table 1:** Crystallite size of CeO_2_ nanostructures.

Catalyst	Crystallite Size at (111) Plane (nm)
CeO_2_-Nps-400	11.3
CeO_2_-Nps-500	21.5
CeO_2_-Nps-600	23.5
CeO_2_-Nrs-400	15.0
CeO_2_-Nrs-500	24.0
CeO_2_-Nrs-600	24.9
CeO_2_-Ncs-400	28.7
CeO_2_-Ncs-500	30.8
CeO_2_-Ncs-600	34.0

**Table 2:** Textural properties of CeO_2_ nanostructures.

Catalyst	BET Surface Area (m^2^/g)	Pore Volume (cm^3^/g)	Average Pore Size (nm)	Crystallite Size (nm)
CeO_2_-Nps-400	66.1	0.275	27.5	11.3
CeO_2_-Nps-500	47.5	0.216	14.0	21.5
CeO_2_-Nps-600	20.2	0.183	13.7	23.5
CeO_2_-Nrs-400	57.8	0.145	24.0	15.0
CeO_2_-Nrs-500	43.9	0.129	20.9	24.0
CeO_2_-Nrs-600	23.0	0.126	10.8	24.9
CeO_2_-Ncs-400	22.5	0.134	17.1	28.7
CeO_2_-Ncs-500	12.6	0.071	8.4	30.8
CeO_2_-Ncs-600	11.1	0.045	6.0	34.0

## Data Availability

Data are contained within the article or Supplementary Material.

## Supplementary Materials

The following supporting information can be downloaded at: https://www.mdpi.com/article/10.3390/molecules28134963/s1, Figure S1. HPLC chromatogram of vanillin standard, Figure S2. HPLC chromatogram of derived sample.
