# Supplementary material for: Direct Oxidation of Hibiscus cannabinus Stalks to Vanillin Using CeO2 Nanostructure Catalysts
Source: Molecules. 2023 Jun 24;28(13):4963. doi: 10.3390/molecules28134963 (PMC10343839; doi:10.3390/molecules28134963)
Supplement: Supplementary file 1 [file molecules-28-04963-s001.zip › molecules-2397800-supplementary.pdf]

molecules

MDPI

Article

Direct Oxidation of *Hibiscus cannabinus* Stalks to Vanillin Using CeO_2 Nanostructures Catalyst.

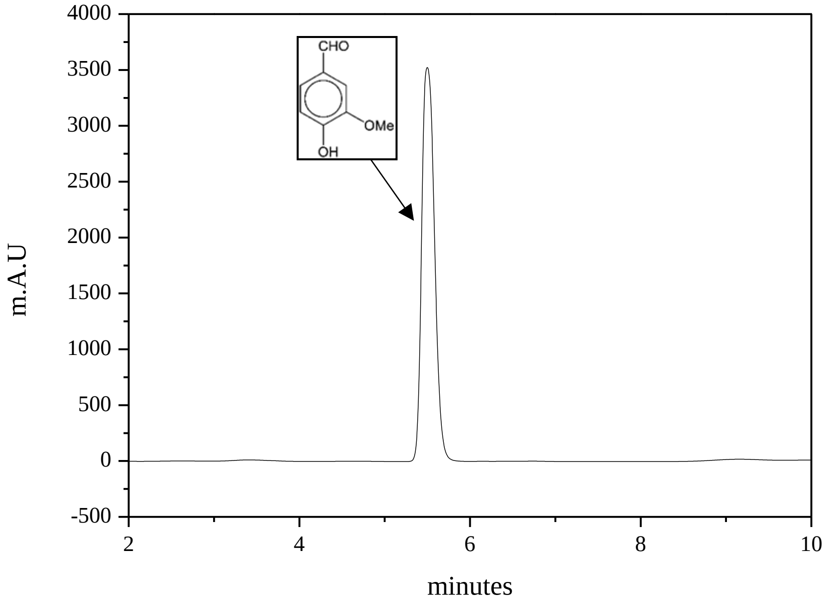

Figure S1. HPLC chromatogram of vanillin standard.

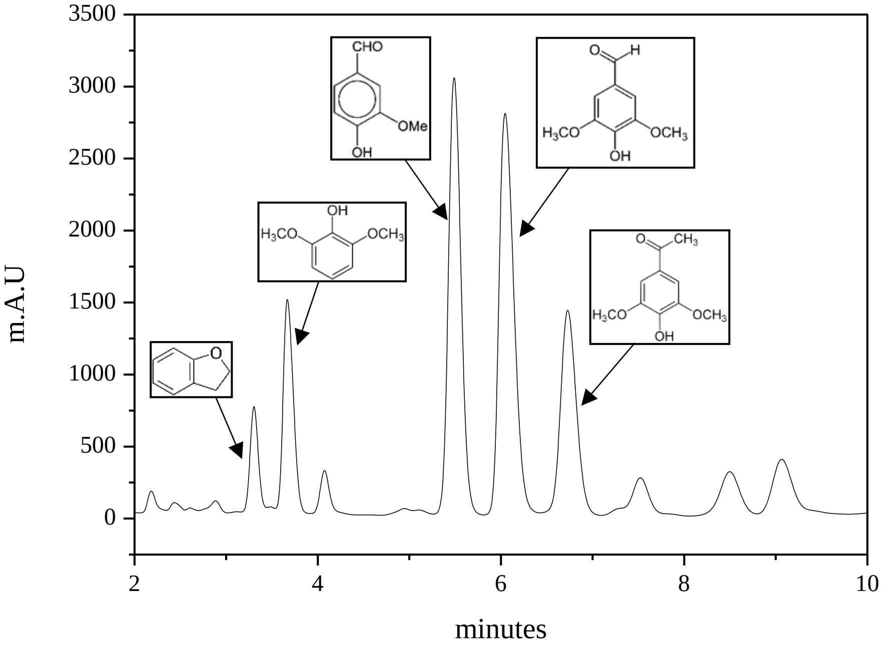

Figure S2. HPLC chromatogram of derived sample.
